# A 3D analysis of growth trajectory and integration during early human prenatal facial growth

**DOI:** 10.1038/s41598-021-85543-5

**Published:** 2021-03-25

**Authors:** Motoki Katsube, Shigehito Yamada, Natsuko Utsunomiya, Yutaka Yamaguchi, Tetsuya Takakuwa, Akira Yamamoto, Hirohiko Imai, Atsushi Saito, Siddharth R. Vora, Naoki Morimoto

**Affiliations:** 1 Department of Plastic and Reconstructive Surgery, Kyoto University Graduate School of Medicine, 54 Kawahara-cho, Shogoin, Sakyo-ku, Kyoto, 606-8507 Japan; 2 Congenital Anomaly Research Center, Kyoto University Graduate School of Medicine, Yoshida-Konoe-cho, Sakyo-ku, Kyoto, 606-8501 Japan; 3 Human Health Sciences, Kyoto University Graduate School of Medicine, 53 Shogoin-Kawahara-cho, Sakyo-ku, Kyoto, 606-8507 Japan; 4 Department of Diagnostic Imaging and Nuclear Medicine, Kyoto University Graduate School of Medicine, 54 Kawahara-cho, Shogoin, Sakyo-ku, Kyoto, 606-8507 Japan; 5 Department of Systems Science, Kyoto University Graduate School of Informatics, Yoshida-Honmachi, Sakyo-ku, Kyoto, 606-8501 Japan; 6 Institute of Engineering, Tokyo University of Agriculture and Technology, 2-24-16 Naka-cho, Koganei-shi, Tokyo, 184-8588 Japan; 7 Oral Health Sciences, University of British Columbia, JBM 372-2199 Wesbrook Mall, Vancouver, BC V6T 1Z3 Canada

**Keywords:** Embryology, Morphogenesis, Intrauterine growth, Anatomy

## Abstract

Significant shape changes in the human facial skeleton occur in the early prenatal period, and understanding this process is critical for studying a myriad of congenital facial anomalies. However, quantifying and visualizing human fetal facial growth has been challenging. Here, we applied quantitative geometric morphometrics (GM) to high-resolution magnetic resonance images of human embryo and fetuses, to comprehensively analyze facial growth. We utilized non-linear growth estimation and GM methods to assess integrated epigenetic growth between masticatory muscles and associated bones. Our results show that the growth trajectory of the human face in the early prenatal period follows a curved line with three flexion points. Significant antero-posterior development occurs early, resulting in a shift from a mandibular prognathic to relatively orthognathic appearance, followed by expansion in the lateral direction. Furthermore, during this time, the development of the zygoma and the mandibular ramus is closely integrated with the masseter muscle.

## Introduction

Growth and development of the facial skeleton is multifaceted and results from a combination and interaction of genetic and epigenetic factors^1^. In very early embryonic stages, specific genes are presumed to regulate the morphogenesis of the facial skeleton. Mutations or gross mis-regulation of such genes cause severe craniofacial congenital anomalies^2^. However, variation in facial skeletal morphology can also be attributed to small, subtle differences in gene regulation amongst individuals. Importantly, such epigenetic regulatory control is exerted by tissues surrounding the facial bones, for example the neural, cartilaginous and muscular tissues^3^. Indeed, such integration of growth in the composite facial region is the basis of Moss’s functional matrix theory, suggesting that “bones do not grow; bones are grown”^3–5^.

It is important to note that by the end of the embryonic period, facial morphology is already recognizable, with individual facial bones attaining a distinct form during early fetal stages^6–9^. Yet, there are very few studies which address these early developmental stages^9^, mainly due to lack of accessibility to appropriate study specimens. Most existing studies have relied on two-dimensional (2D) histological sections or x-rays, and have used simple linear measurements in their analyses^8,10,11^. These traditionally used 2D images and linear morphometrics, pose several limitations in quantifying and understanding the complex, three-dimensional (3D) facial skeleton. Geometric morphometrics (GM) utilizes 3D landmark coordinates, retains geometric information and applies multivariate statistics, hence enabling comprehensive quantification and visualization of shape differences between objects. GM analysis also allows for a comprehensive study of allometry, which represents the change in shape accompanying a change in size (or age) and is an important element of facial growth. The most dramatic shape changes in the facial skeleton has been reported to occur before the second trimester, when the facial skeletal elements have not yet fully mineralized into bone^6,8–10,12^ and cannot be detected by CT scans. Magnetic resonance imaging (MRI) is hence an appropriate and advantageous tool for the detailed 3D-analysis of the early fetal facial skeletal morphogenesis^6,12^. Additionally, MRI analysis allows for a clear visualization and assessment of the developing facial musculature.

The functional relationship between masticatory muscles and their influence on facial skeletal shape is an intriguing aspect of facial growth, and has been the focus of several studies^13–20^. Reduced masticatory requirements have been proposed to impact evolutionary changes in the facial shape of modern human, such as attainment of a more orthognathic profile, narrower face, and smaller mandible^21^. A significant influence of masticatory forces on facial skeletal shape has been determined in experimental studies using botulinum toxin^22^ and dietary modifications^23^, as well as morphometric studies inferring mastication force by quantifying muscle cross-sectional area (CSA)^13,16^. However, the relationship between muscles of mastication and primary facial morphogenesis during the prenatal period, is unclear. Although true mastication is not occurring in the fetus, significant movements of the tongue, mouth opening, neck flexion and swallowing of amniotic fluid can be seen early during development. These movements result from the action of masticatory and other muscles attached to various facial bones. In individuals with fetal akinesia deformation sequence (FADS), a neural dysfunction in the fetal period which results in the lack of muscle activity; associated hypoplasia of the zygoma and the mandible has been noted^24^. This suggests the presence of integrated growth between facial bones and attached musculature, at these early developmental time points.

An essential component of studying facial growth is the positional relationship between the midfacial skeleton and the mandible. Once sufficient teeth have erupted into the oral cavity, a fairly reproducible mandibular occlusal position can be assessed. In adults, this is routinely obtained from a standard cephalometric radiograph. However, in embryos and fetuses, this position cannot be established due to the lack of erupted teeth. Hence, a mouth-open position of a fetus may reflect a passive position of the mandible at the time of sample collection, which may have been produced either naturally due to fetal movements (as described above), or artificially at the time of collection. Either way, this position may not be a true representation of the mandibular-maxillary relationships and if morphological analyses are performed as-is, this mouth-open position will be captured incorrectly as morphological variation. Therefore, standardizing the mandibular position prior to conducting morphological analysis is beneficial, in order to understand the skeletal development and positional changes more accurately.

To quantify and visualize the ontogenetic growth allometry of the human facial skeleton during the early fetal period, we used high-resolution 3D MR images of a total of 49 human embryo and fetuses, and GM analyses with a focus on non-linear estimation of the growth trajectory. Notably, we corrected the mouth-open position in our analysis, to enable the assesment of a more appropriate relationship between the mandible and midfacial bones. Additionally, we investigated the relationship between the developing skeletal units of the face (zygoma and mandible) and associated muscles of mastication (masseter and temporalis), in order to study the integration between these structures early during human development. Our results suggest that the facial skeleton displays dramatic allometry, with distinct periods of combined anteroposterior and lateral growth, as the midfacial and mandibular skeletal segments begin to take shape. The lateral portion of the facial skeleton, including the zygoma and the ramus of the mandible, transforms significantly in the fetal period, and their shape changes are highly integrated with the development of masticatory muscles, mainly the masseter.

## Material and methods

### Specimens and image acquisition

Since 1961, a large number of human conceptuses have been collected and maintained at the Congenital Anomaly Research Center at Kyoto University (Kyoto Collection)^25,26^. Most of these specimens were collected after artificial abortion, in accordance to the Maternity Protection Law of Japan. The study was approved by the Ethics Committee at Kyoto University Graduate School and Faculty of Medicine, Kyoto, Japan (R0316, R0347, and R0989). Only specimens without any distinct congenital anomalies and artificial deformities of the face were selected for the present study. We initially obtained MR images of a total of 92 specimens, including 5 embryos and 87 fetuses, using a 7-T MR system (Biospec 70/20 USR; Bruker Biospin MRI GmbH, Ettlingen, Germany) and a 3-T MR system (MAGNETOM Prisma; Siemens Healthcare, Erlangen, Germany). We excluded 43 specimens either due to a distortion, a defect in the facial skeleton, or artifacts in the MR images. Consequently, a total of 49 specimens, including 1 embryo and 48 fetuses, with CRL ranging from 29.8 to 225 mm, were used for this study. The sex of all specimens was determined based on observation: 21 were male, 20 were female, and 8 were indistinguishable (Supplementary Table 1). All parents of these specimens were Japanese. Gestational age (GA) was calculated according to the CRL using Sahota’s equation ($GA=26.643+7.822\times \sqrt{CRL}$)^27^.

### Landmark annotation, cross-sectional area (CSA) calculation and surface model generation

A total of 44 landmarks were digitized on the MR images of the facial skeleton using Checkpoint software (Stratovan, Davis, CA, USA), including 29 landmarks on the midface and 15 landmarks on the mandible (Supplementary Fig. 1 and Supplementary Table 2). These landmarks were chosen based on previous studies^6,7,12,17,19^. Landmark coordinates were exported to MATLAB 9.0.1 (Mathworks, Natick, MA, USA) for multivariate statistical shape analyses.

**Figure 1:**
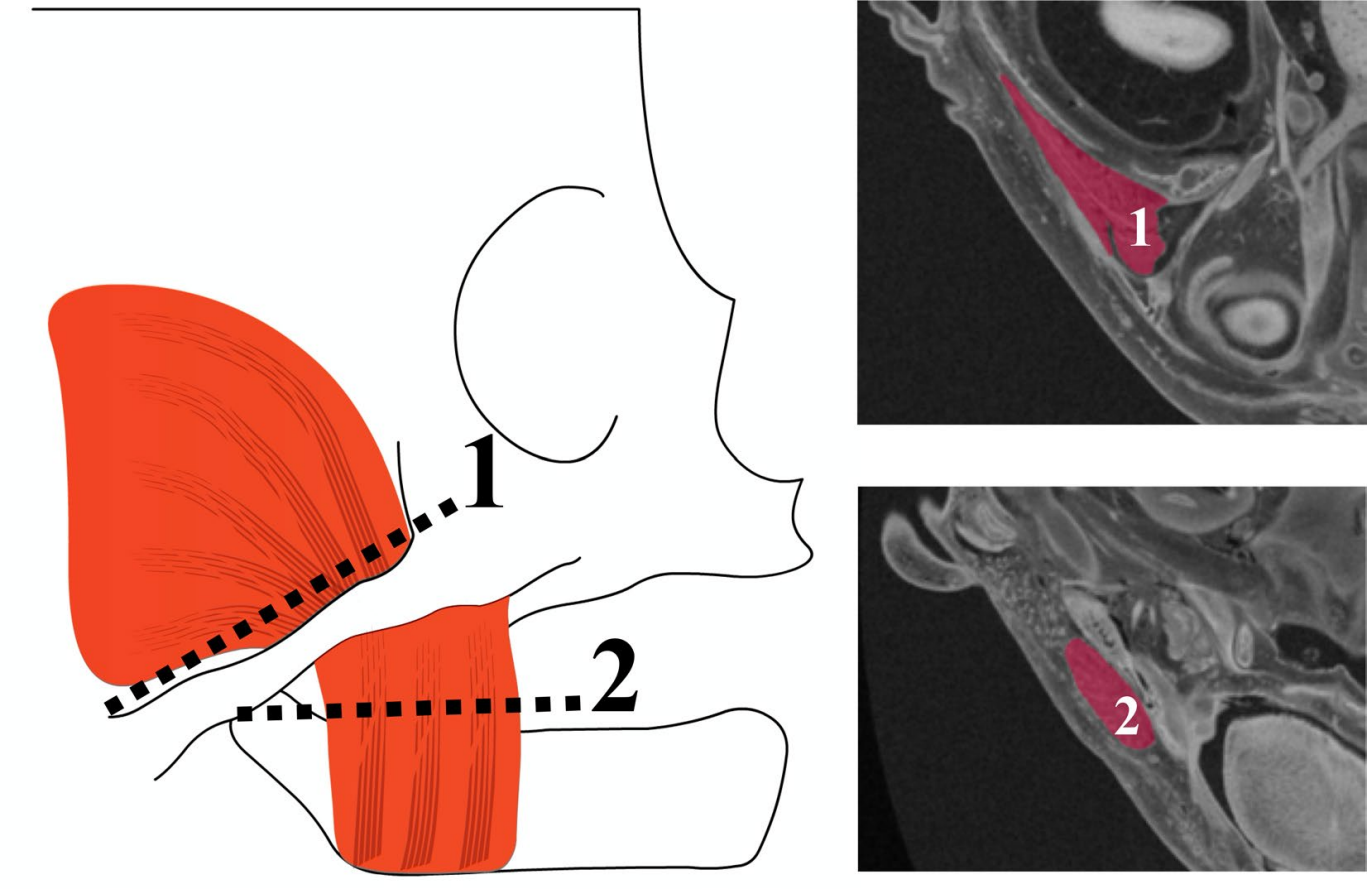
Measurement of muscle cross-sectional areas. The cross-sectional area (CSA) of the temporalis and masseter muscles were measured from MRI slices obtained at the level of a plane placed parallel and just superior to the zygomatic arch (1) and a plane parallel to the mandibular plane (2), respectively.

The cross-sectional areas (CSA) of the masseter and temporalis muscles were measured using Horos software (version 3.3.6, the Horos Project, Annapolis, MD, USA). Location of the plane for measuring the CSA was modified based on Weijs and Hillen^13^. For the temporalis muscle, a plane parallel and just superior to the horizontal part of the zygomatic arch was selected (Fig. 1, #1). For the masseter muscle, a plane parallel to the mandibular plane and just superior to the mandibular body was selected (Fig. 1, #2)^15,16^. The area occupied by the muscles in these planes was manually segmented twice by same investigator (M.K.), 4 weeks apart, to evaluate the reproducibility of the CSA^16^. Intraclass correlation coefficient (ICC) analysis using a one-way random effects model^28^ was carried out in R 3.4.1 (R Core Team, 2017, library *irr*^29^). CSA measurements were highly reproducible for both the masseter and temporalis muscles (ICC = 0.999). The average score of the two of CSAs for each specimen was used in this study. All the specimens were analyzed bilaterally. Eight specimen had poor visualization of muscles in MR images on one side and were hence excluded. A total of 90 sides from 49 specimens were included in this study.

For visualization of shape analysis, a manual segmentation of the craniofacial bones from one specimen (CRL 86 mm) was performed using Amira software (version 6.0.1; Visualization Sciences, Berlin, Germany), followed by a surface model generation.

### Correction of mouth-open position

**Figure 2:**
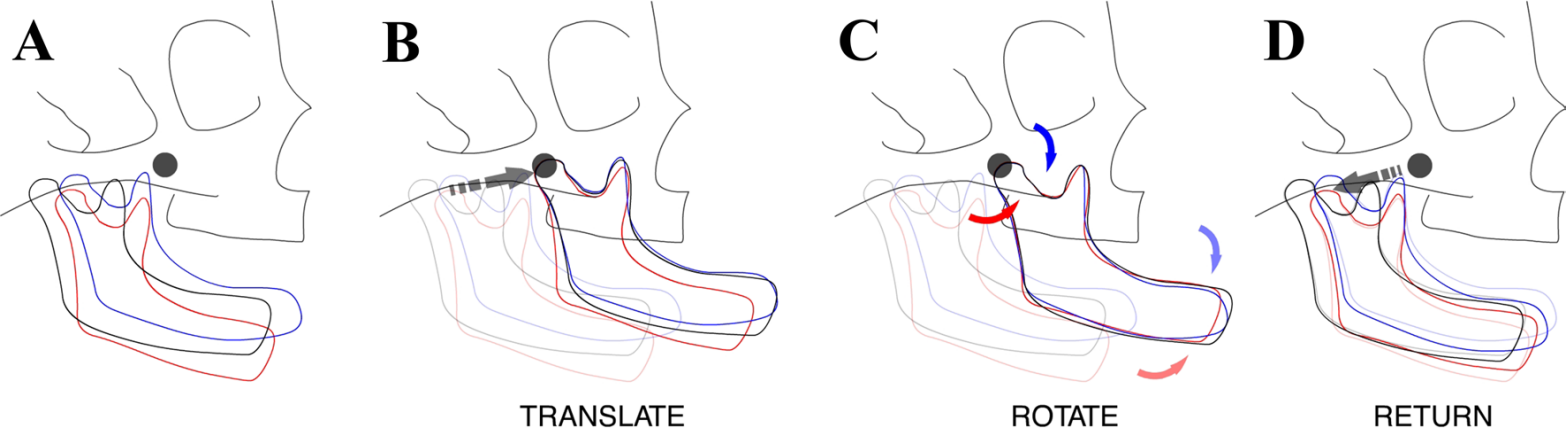
Standardization of open-mouth position of mandible. Figure depicts the series of steps performed to standardize the open-mouth position of the mandible (see Materials and Methods). Briefly, a generalized Procrustes superimposition was performed to optimally register all specimen at the centroid (**A**, grey dot). The complete set of mandibular coordinates only, was translated such that the mid-condylar point for each specimen coincided with the centroid of the overall superimposition (**B**). Mandibular coordinates of each specimen were then rotated around its condylar axis, to find the best rotation degree which minimized the Procrustes distance between its configuration and that of the mean mandibular configuration (**C**). Finally, the rotated set of the mandibular coordinates of each specimen were translated back so that the mid-condylar points returned to their original positions, while maintaining the rotation (**D**). These new set of mandibular coordinates were recombined with the Procrustes coordinates of the midface.

Based on the observation on the mid-sagittal plane of the MR images, the tongue closely approximated the palate, and the lips were in contact in most of our specimens. However, several specimens did not display such contacts and had a slight mouth-open position (Supplementary Fig. 2). In fact, our initial estimation of growth allometry using the results of a generalized Procrustes superimposition (see below), followed by a principal component (PC) analysis and the multivariate regression of PCs on the CRL^6,7^ demonstrated a gradual opening of the mouth, with increasing CRL (Supplementary movie 1). Although, some of this mouth-opening may reflect a true ontogenic morphological trend, some of it could be due to fetal jaw movements, which have been reported to begin as early as 11 weeks of gestation^30^, or artificial opening during specimen collection and storage (Supplemental Fig. 3). Hence, we first accounted for this mouth-open position in our specimen by performing a set of transformations focused around the bilateral condylar head landmarks (Supplementary Table 2, Landmark #39 and #40) for each specimen. The axis connecting these landmarks is referred to as the condylar axis and the midpoint of these landmarks is referred to as the mid-condylar point (MCP). First, to obtain an optimal registration of all the landmarks used in the study, a generalized Procrustes superimposition was performed. This ensured that the landmark coordinates were translated, scaled, and rotated to the best-fit superimposition, and yielded new- Procrustes coordinates for each specimen and also allowed for a mean shape calculation. Next, the complete set of mandibular Procrustes coordinates were separated from the rest of the Procrustes coordinates of the midface. The set of mandibular coordinates was then translated such that the MCP for each specimen coincided with the centroid of the overall superimposition. Next, the mandibular coordinates of each specimen were rotated around its condylar axis, in order to find the best rotation degree which minimized the Procrustes distance between its configuration and that of the mean mandibular configuration. Next, the rotated set of the mandibular coordinates of each specimen were translated back so that the MCP returned to its original position, while maintaining the rotation. These new set of mandibular coordinates were finally recombined with the Procrustes coordinates of the midface (Fig. 2). A second generalized Procrustes analysis was then performed on the adjusted coordinates. This final set of coordinates had the optimal superimposition as well as a standardized mouth position, and were utilized for all downstream analyses.

### Estimating and visualizing the non-linear growth trajectory

The growth trajectory was calculated using weighted average analysis (Nadaraya–Watson kernel regression)^31,32^ using the Procrustes coordinates obtained after adjusting for the mouth-open state^33,34^ (see above). Leave-one-out cross validation was performed to optimize kernel widths $\sigma$. In this procedure, the error ${\sum }_{i}{\Vert {x}_{i}-{f}_{i}\left({t}_{i}\right)\Vert }^{2}$ was minimized, where ${x}_{i}$ and ${t}_{i}$ are the feature vector and the time point for the $i$-th specimen, respectively, and ${f}_{i}$ is the regression curve estimated from the feature vectors except for ${x}_{i}$. Note that, due to the boundary problem of the kernel regression, the error was calculated only for those specimen with ages between 10 and 90th percentiles (i.e. specimens with CRL between 35.4 to 186 mm, which were between approximately 10.5 to 19 weeks of gestation). The shift of the landmarks along the estimated growth trajectory was obtained and the expected shape at each time point was visualized by warping the generated surface model of a template specimen according to the shift of landmarks using a radial basis function interpolation^7,12,35^. In other words, homologous landmarks on the template specimen, along with all neighboring mesh of the generated surface model were artificially “stretched” to fit the landmark shifts obtained from the growth trajectory analysis. Next, a PC analysis was performed to visualize the growth trajectory in PC1-3 spaces, and a bootstrap procedure (1000 resampling) was carried out to compute 95% confidence regions. To determine periods when the growth trajectory changed, the curvature of the trajectory was computed by fitting a circle to it and then calculating a curvature for each triplets of consecutive points, densely sampled on the trajectory.

Sexual dimorphism was assessed using a MANOVA analysis, testing PCs 1–19 which explains > 90% of the overall shape variation in our specimen (Supplementary Table 3). Since sex was not recognized as a significant co-variant in the MANOVA, all the specimens were used together for the growth trajectory analyses.

### Growth integration between masticatory muscles and facial bones

Five landmarks were assigned to the zygoma (Supplementary Table 2, #), while five landmarks were assigned to the ramus of the mandible (Supplementary Table 2, *). Since the Procrustes coordinates used in this study did not include size, the CSA for each specimen was also adjusted for size. This was achieved by divided the CSA for each specimen by the square of its CRL—referred to CSA index (CSAi). To investigate the developmental integration between mastication muscles and associated bones, a two-block partial least-squares (PLS) analysis was performed with R 3.4.1 (R Core Team, 2017) using the library geomorph^36,37^. The CSAi were entered singly into the analysis as one ‘block’ of data and landmarks of the zygoma or the ramus of the mandible as the other block^15^. The PLS analysis yielded a singular warp including the maximum possible covariance^38,39^. To quantify the strength of the correlation between the CSAi of the temporalis and masseter muscles and the shape of the zygoma and the ramus of the mandible, we calculated Pearson’s product moment correlation coefficient (r) and the RV coefficient^40^. The RV coefficient, which is analogous to Pearson’s R^2^, represents the level of integration and measures the proportion of the total variance explained by the covariance of two blocks^41,42^. The P-values of PLS analyses were calculated using permutation tests with 10,000 resamples. The permutation test was performed in the way that the CSAi and shape block was permuted and PLS-scores recomputed for each permutation. The threshold of significance was set at *P* = 0.01.

A radial basis function interpolation was utilized to warp the surface models of the zygoma and the mandible with MATLAB 9.0.1, in order to visualize the shape changes along their singular warping, which represents the axis of covariation of these bones to CSAi of the masseter muscle.

### Data availability

The datasets analyzed during the current study are available from the corresponding author on request.

## Results

### Growth trajectory of facial skeleton during early fetal stages

**Figure 3:**
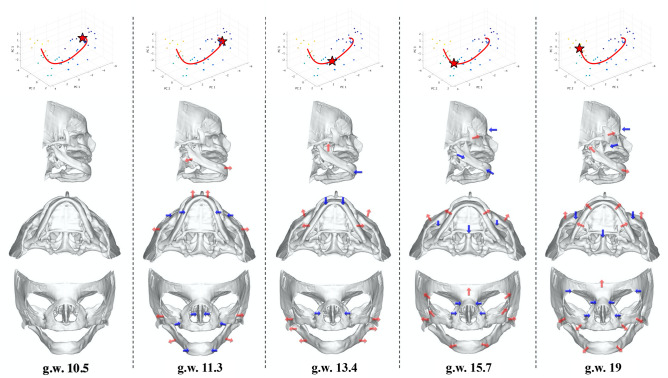
Facial skeletal morphogenesis along the growth trajectory. The PC plots in the top row depicts the distribution of specimens along PCs 1, 2 and 3. Colored dots represent individual specimen (blue = youngest and yellow = oldest) and the 3 dimensional red line indicates the growth trajectory from youngest to oldest. Columns 1–5 depict the skull shape changes from an infero-lateral, inferior and frontal views (2nd, 3rd and 4th rows respectively). The “red star” in each PC plot corresponds to the estimated gestation week during which significant changes in facial shape can be noted, as represented by the bend in the red line. Red arrows indicate relative outward and forward growth, while blue arrows indicate relative inward in backward growth in specific areas of the fetal skull.

The growth trajectory was estimated using a non-linear weighted average analysis (Nadaraya–Watson kernel regression)^31,32^ followed by a PC analysis. The first three PCs accounted for 38.6%, 9.7%, and 7.9% (cumulatively 56.2%) of the total variance. A bootstrap resampling was performed to calculate 95% confidence intervals (Supplementary Fig. 4). The trajectory had 3 bending points (Fig. 3, top row, red stars), indicating that the facial morphology changes could be viewed as 4 unique segments, during this age period. The curvature of the growth trajectory was computed, yielding the timings of these bending points. Based on the CRL of specimens (44.8, 73.2 and 112.6 mm), these changes were estimated to occur at 11.3, 13.4 and 15.7 weeks of gestation (Supplementary Fig. 5).

To visualize the facial skeletal morphogenesis along the growth trajectory, the generated surface model of the template specimen was warped using a radial basis function interpolation, based on the shift of landmarks obtained at each time point (Fig. 3 and Supplementary Movie 1)^7,12,35^. During the first growth segment (10.5–11.3 weeks of gestation), the lateral part of the facial skeleton expanded, resulting in the appearance of a relative reduction in the width of the nasal cavity, maxilla, and anterior mandible. At the same time, a relative antero-posterior enlargement occurred in the maxilla and the mandible, more pronounced in the latter, resulting in the appearance of mandibular prognathism. During the second growth segment (11.3–13.4 weeks of gestation), the facial appearance changed from being mandibular prognathic to relatively orthognathic, with the naso-maxillary complex showing a more pronounced antero-posterior expansion relative to the mandible. Concomitantly, the mandibular width increased relatively at its proximal end, around the gonial angles, and the zygoma developed in the antero-lateral dimension. The lateral expansion of the mandible and the antero-lateral development of the zygoma continued into the next segment (13.4–15.7 weeks of gestation). During the last segment (15.7–19 weeks of gestation), minor shape changes occurred, with the zygoma continuing to develop antero-laterally and the mandible showing marked lateral development in the body and ramus areas (Fig. 3 and Supplementary Movie 1).

### Integration between developing masticatory muscles and associated facial bones

**Figure 4:**
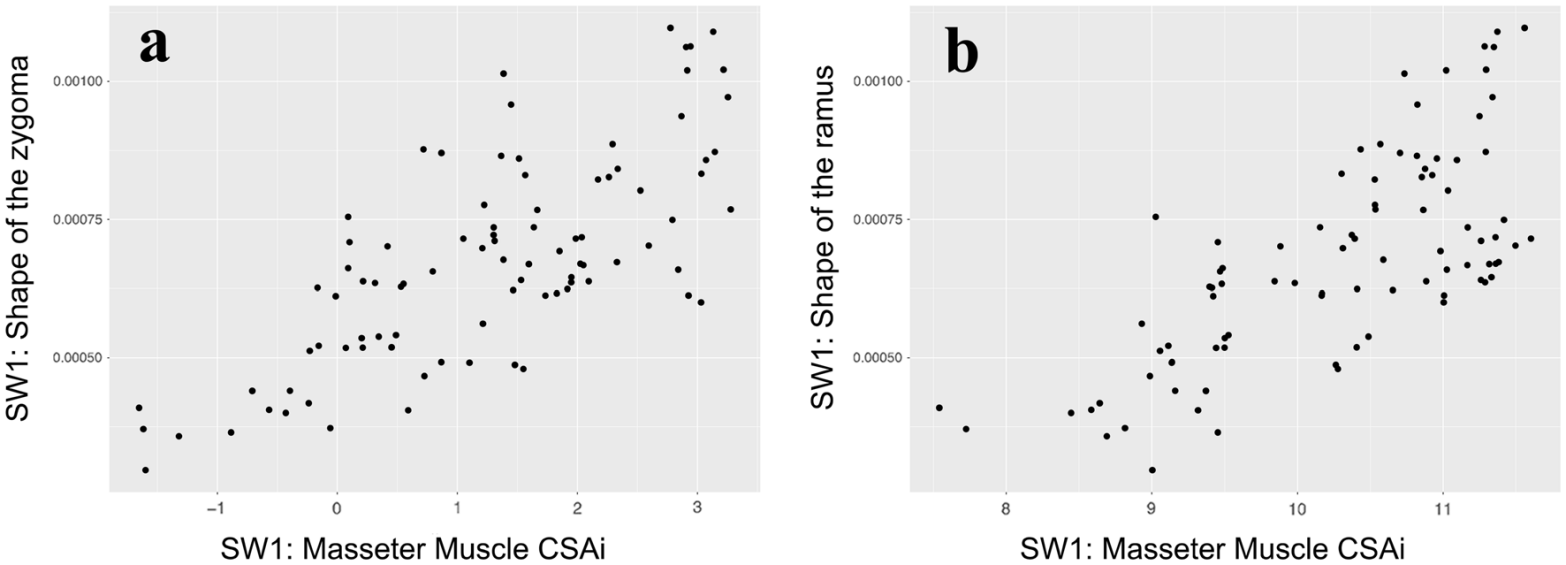
Masseter muscle—facial bone integration. Plots of singular warp (SW1) of the CSAi of the masseter muscle (x-axis) against SW1 of the shape of the zygoma (**a,** y-axis) and the ramus of the mandible (**b,** y-axis). Scores on these axes are significantly correlated (r = 0.737; RV = 0.506; *P* = 0.0001; 95% confidence interval = 0.625, 0.819 and r = 0.725; RV = 0.408; *P* = 0.0001; 95% confidence interval = 0.609, 0.81, respectively).

**Figure 5:**
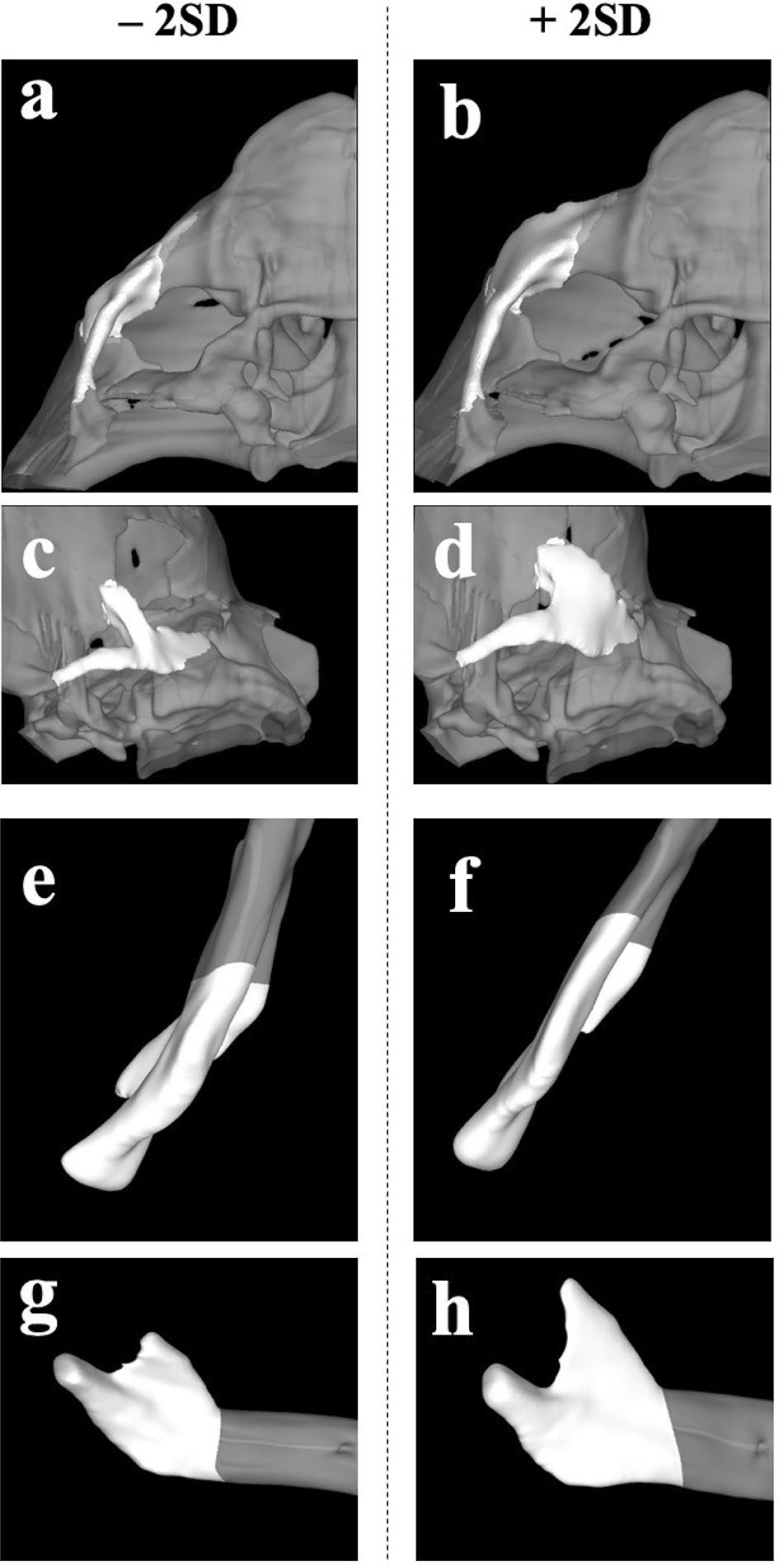
Change in shape in the zygoma and mandibular ramal areas correlated to masseter muscle growth. The shape changes seen in the zygoma (**a**–**d**) and the ramus of the mandible (**e**–**h**) along the first singular warp (SW1), on the cross-sectional areas (CSAi) of the masseter muscles from − 2 (**a**,**c**,**e**,**g**) and + 2 (**b**,**d**,**f**,**h**) standard deviations (SD). The body of the zygoma appears to expand in an antero-lateral direction with increasing masseter CSAi (**a**,**b** Inferior view; **c**,**d** Lateral-inferior view). The width of the ramus and the coronoid process appears to expand, and the mandibular body shifts laterally with increasing masseter CSAi (**e**,**f** inferior view; **g**,**h** lateral view).

A two-block PLS analysis was performed to evaluate the developmental integration between masticatory muscles (masseter and temporalis), and associated facial bones, such as the zygoma and the ramus of the mandible. The RV coefficient was calculated as a measure of association between these blocks. A significant correlation was found between the midface and mandibular shape change, and CSAi of the masseter and temporalis muscles (Table 1, Fig. 4). The CSAi of the masseter muscle had a strong, statistically significant correlation with the zygoma (r = 0.737; RV = 0.506; *P* = 0.0001; 95% confidence interval = 0.625, 0.819) and the ramus (r = 0.725; RV = 0.408; *P* = 0.0001; 95% confidence interval = 0.609, 0.81) shape changes. The transformation along the singular warp indicated that the body of the zygoma expanded in an antero-lateral direction with increasing masseter CSAi (Fig. 5, a-d and Supplementary Movie 2). At the same time, the width of the ramus and the coronoid process expanded, and the mandibular body shifted laterally (Fig. 5e–h and Supplementary Movie 3). The CSAi of the temporalis muscle had a very week correlation with the zygoma (r = 0.507; RV = 0.0528; *P* = 0.0002) and the ramus (r = 0.42; RV = 0.0862; *P* = 0.0047) shape changes.

**Table 1:** Integration between facial skeleton and masticatory muscle CSAi.

	Masseter CSAi			Temporalis CSAi		
	Correlation (r)	RV	*P *value	Correlation (r)	RV	*P *value
Zygoma	0.737	0.506	0.0001	0.507	0.0528	0.0002
Ramus	0.725	0.408	0.0001	0.42	0.0862	0.0047

## Discussion

This study focuses on the ontogenetic allometry (shape change as a function of development) of the facial skeleton during the prenatal period, with a specific focus on epigenetic influences from surrounding muscles^1^.

Given the highly complex nature of facial skeletal growth, the direction of allometric changes are not straightforward. Consequently, while conventionally used linear estimations of allometry are fairly effective, non-linear estimation of the allometry can represent the intricacies of facial growth trajectories much more appropriately. Matthews et al.^43^ modelled such non-linear growth trajectory to study facial growth and sexual dimorphism in children and adolescents patients. We applied similar non-linear allometry assessment to explore early prenatal craniofacial development.

In adults, a fairly standardized and reproducible mandibular position can be determined when teeth are in maximum intercuspation, establishing an occlusal stop to mandibular closing. But in developing fetuses or early infants, what determines such a standardized mandibular position? In the early prenatal period, tongue development is closely associated with the palatal formation^44–47^ and the vertical position of the mandible. Thus, the mandibular position where the tongue contacts with the palate could be considered as a determinant of a standardized position. The other determinant could be lip closure. However, these soft tissue determinants are not enough to firmly define the hard tissue position. In our specimen, the trend of tongue contacting palate or lip closure was varied, and did not relate to CRL (Supplementary Table 1, Supplementary Fig. 2). This may be either due to fetal jaw movements, which have been reported to begin as early as 11 weeks of gestation^30^, or artificial opening during specimen collection and storage. Indeed, in our initial PC analysis, mouth-open was identified as a major morphological change occurring with increased CRL (Supplementary Movie 4). Therefore, to account for these possible artifacts on overall facial morphogenesis, our analysis was performed after standardizing the mouth-open state, for all the specimens, in line with similar data processing performed in other studies^48^.

Results from our study indicate that the human fetal facial profile is mandibular prognathic until ~ 11.3 weeks of gestation. Similar to our findings, Humphrey^30^ reported that the human fetal facial profile was most prognathic at ~ 11.5 weeks of gestation, following which, mandibular growth lagged behind the midface until about 20 weeks of gestation^30,49^. Similarly, Diewert^9,50^ reported that at the end of embryonic period the facial profile appeared mandibular prognathic and then reverted back to an orthognathic profile ~ 12 weeks of gestation, concluding that the slower elongation of the mandible corresponded to a transitional period during which the bony mandible with its secondary cartilages, replaced Meckel’s cartilage as the major skeleton of the lower jaw. Katsube et al.^7^ reported that the mid-facial skeleton developed dramatically in the antero-posterior dimension until about 14 weeks of gestation. Combining our current findings with what is known, it appears that the acquisition of a stable maxillo-mandibular relationship results from a more rapid anterior–posterior growth of the midface, and a slower anterior growth of the mandible, sometime after the 11th week of gestation.

Coquerelle et al.^51^ investigated facial growth allometry using 20 human fetuses ranging from 10–34 weeks of gestation and digitized landmarks on the midfacial skeleton and the mandible. Consistent with our results, they showed that the mandibular body became relatively short and its morphology changed from being straight and “V”-shaped, to slightly rounded and “U”-shaped. However, their growth trajectories indicated a combination of different linear growth processes in the PC space, with 2 transitional periods at 16- and 25-weeks of gestation. Additionally, their results also showed that the maxillo-mandibular relationship changed from prognathic to relatively orthognathic before 16 weeks of gestation. Although this pattern is consistent with what we report (Fig. 3 and Supplementary Movie 1), in our analysis, this switch occurs around 11.3 weeks. A few factors that may account for this difference include a larger sample size in our study, the adjustment of the mouth-open state, and a purely Japanese population from which our specimen were derived.

There is evidence of strong developmental and functional integration between growth of the human facial skeleton and its surrounding structures. These structures are mainly the neural/sensory organs contained within the craniofacial skeleton, cartilaginous growth centers and various oral, pharyngeal, facial and masticatory muscles. With regards to these relationships during the prenatal stages of development, the impact of cartilaginous growth centers on facial bone growth seems applicable, given the strong similarity of these skeletal tissues. For e.g. Katsube et al.^7^ investigated the integration between growth centers, such as the nasal septum and spheno-ethmoidal synchondrosis with mid-facial growth, in the early prenatal period, demonstrating that the growth centers were closely associated with antero-posterior growth of the mid-facial skeleton. However, the relationships between muscle and bone growth during this early developmental period, has not been well documented.

The attainment of an orthognathic facial profile in modern humans has been related to the range of masticatory forces^21^, with evolutionary trends towards narrower faces and high mandibular plane angles when compared to ancestors. At the population level, it has been proposed that individuals with high masticatory forces tend to have wider faces with a pronounced gonial, mandibular angle and vise versa^52^. Many earlier studies have found a positive correlation between facial width and the muscle CSAs, especially that of the masseter muscle^13,53,54^. However, these studies have traditionally relied on linear measurements. Sella-Tunis et al.^16^ and Toro-Ibacache et al.^15^ applied 3D GM methods using PLS analysis to study the correlation between masticatory muscles and the mandibular shape or mid-facial skeletal shape in adults, respectively. Sella-Tunis et al.^16^ found that higher masseter and temporalis muscle CSAs associated with a wide trapezoidal shaped ramus, large coronoid process, and a curved basal arch in adults. Toro-Ibacache et al.^15^ found that a larger temporalis correlated with enlargement of the temporal fossa and suggested such changes in fossa size may provide space for muscles with large CSAs. Ours is the first study which focuses on the interactions between muscular and skeletal relationships in the early prenatal period. Our results show that the developing masseter muscle already closely correlates to the shape of facial skeletal components, specifically an antero-lateral expansion of the zygoma and the lateral development of the ramus of the mandible, in the early prenatal period (Fig. 5).

Humphrey^30^ suggested that a distinct mouth opening in fetuses followed by a reflexive jaw closure begins as early as ~ 11 weeks of gestation (CRL > 55 mm) and occurs primarily through the action of the masseter and temporalis muscles. Our growth trajectory results indicate that the lateral portion of the facial skeleton, such as zygoma or the ramus of the mandible, transforms slowly but significantly, until 19 weeks of gestation. Thus, masticatory muscle activity during the early fetal period is likely an important factor for morphogenesis of the lateral facial skeleton, even in the second trimester of pregnancy. Indeed, this may explain the unique facial skeletal abnormalities associated with fetal akinesia deformation sequence (FADS). FADS originates as a muscle-function abnormality during the prenatal period. The craniofacial features of FADS includes ocular hypertelorism, high bridge of the nose, underdeveloped tip of the nose, posteriorly angulated ears that appear low set, and microretrognathia (a posteriorly placed small jaw) often with small mouth and/or limited jaw opening, caused by the hypoplasia of the zygoma or the mandible^24^. Given the association we observe between the muscle CSAs and bony growth at this early stage, it is possible that the neurogenic or myopathic disorders in the muscles of mastication associated with FADS, concomitantly results in the observed bony hypoplasias.

When available, longitudinal data are most appropriate for studying craniofacial ontogeny. However, it is very difficult to obtain such data in the early prenatal period, particularly if we want high resolution images where both muscle and bone structure can be accurately recorded. Hence, we have to rely on cross-sectional data, wherein larger sample sizes are desirable. In this regard, a sample size of 49 used in our study is relatively large when compared to similar studies using human specimen in this age group^8,51,55^. Additionally, for precise investigations of ontogeny, reliable assessment of a specimen’s age is also important. Fetal age in prenatal period is commonly represented as gestational age (GA), which is often based on the self-reported menstrual dates in medical records. However, these dates can be unreliable^56,57^ and can hence obscure data analysis if used as a proxy for the specimen’s age. Hence, many studies rely on the CRL measurements of specimen, to establish GA^6,7,10,12,30,35,46,47^. Different equations have been developed to relate GA to CRL. Napolitano et al.^58^ conducted a systematic review in which they assessed 29 different studies proposing varying GA—CRL equations. The equation used in our study (Sahota et al.^27^) was found to be one of four studies with the highest methodological quality and hence more reliable in its estimation of GA.

The growth allometry revealed here is based on the investigated specimens derived from a purely Japanese ancestry. Hence we would caution against applying these results to all human populations. Indeed, distinct population level differences in facial morphology have been identified in several studies, with individuals of Asian descent displaying relatively wider faces, with a distinct mandibular (gonial) angle^59–61^. Although the timing at which population-based facial morphological differences are attained has not been clarified, our results suggest that initiation of some of these aforementioned characteristics may in fact, occur in the early fetal period. Further investigations using multiple sample collections from different geographic locations are desirable, to help clarify the timing of initiation of facial morphological differences amongst different populations.

Our analysis reveals intriguing ontogenic changes in the different facial skeletal components, which accompany the early attainment of the relatively orthognathic profile in human faces. The facial skeleton displays dramatic allometry, with distinct periods of combined anteroposterior and lateral growth, as the midfacial and mandibular skeletal segments begin to take shape. The lateral portion of the facial skeleton, including the zygoma, transformed significantly in the fetal period, and their shape changes correlated strongly with the development of masticatory muscles, mainly the masseter. At these early stages of fetal development, genetic determinants of growth have been presumed to predominate environmental and epigenetic influences. Yet, early fetal movements coupled with the high levels of correlation between the development of masticatory muscles and facial bone growth found here, warrant further attention to early epigenetic influences on bone growth. A previous study demonstrated that the facial skeleton displays slight but significant levels of directional and fluctuating asymmetry in early fetal periods^12^. Although the reasons for this are unclear, this finding suggests that asymmetry could presumably be enhanced by epigenetic influences from functional determinants, such as slight differences in muscle growth between the two sides. Nevertheless, our findings highlight the continued importance of studying facial growth during the fetal periods, not just at the level of mineralized hard tissues of the skeleton, but also the surrounding soft tissues.

## Supplementary Information

Supplementary Video 1.

Supplementary Video 2.

Supplementary Video 3.

Supplementary Video 4.

Supplementary Information 1.

Supplementary Information 2.
