# Supplementary material for: A 3D analysis of growth trajectory and integration during early human prenatal facial growth
Source: Sci Rep. 2021 Mar 25;11:6867. doi: 10.1038/s41598-021-85543-5 (PMC7994314; doi:10.1038/s41598-021-85543-5)
Supplement: Supplementary file 5 — Supplementary Information 1. [file 41598_2021_85543_MOESM5_ESM.docx]

The file ‘Supplementary data’ contains Supplementary figure 1 to 5, Supplementary table 1 to 3, and Supplementary text 1.

**Legends of supplement movies**

**Supplementary movie 1 Shape change along the non-linear growth trajectory**

During the first growth segment (10.5–11.3 weeks of gestation), the lateral part of the facial skeleton expanded, resulting in the appearance of relative reduction in the width of the nasal cavity, maxilla, and anterior mandible. At the same time, a relative antero-posterior enlargement occurred in the maxilla and the mandible, more pronounced in the latter, resulting in the appearance of mandibular prognathism. During the second growth segment (11.3–13.4 weeks of gestation), the facial appearance changed from being mandibular prognathic to relatively orthognathic, with the naso-maxillary complex showing a more pronounced antero-posterior expansion relative to the mandible. Concomitantly, the mandibular width increased relatively at its proximal end, around the gonial angles, and the zygoma developed in the antero-lateral dimension. The lateral expansion of the mandible and the antero-lateral development of the zygoma continued into the next segment (13.4–15.7 weeks of gestation). During the last segment (15.7–19 weeks of gestation), relatively minor shape changes occurred, with the zygoma continuing to develop antero-laterally and the mandible showing marked lateral development in the body and ramal areas.

**Supplementary movie 2 Shape change of the zygoma along singular warp with the cross-sectional area of the masseter muscle.**

The body of the zygoma expanded in an antero-lateral direction, with increasing masseter cross-sectional area.

**Supplementary movie 3 Shape change of the ramus of the mandible along singular warp with the cross-sectional area of the masseter muscle.**

The width of the ramus and the coronoid process expanded, and the mandibular body shifted laterally, with increasing masseter cross-sectional area.

**Supplementary movie 4 Shape change along the axis of the multivariate regression of principal components on the crown-rump length.**

Prior to adjusting the open-mouth position, we performed a principal component (PC) analysis on the facial skeleton. Next, the linear growth allometry was estimated using multivariate regression of PCs on the crown-rump length (CRL). The growth allometry results demonstrated with increasing CRL, the samples gradually displayed an opening of the mouth.
