# Supplementary material for: A 3D analysis of growth trajectory and integration during early human prenatal facial growth
Source: Sci Rep. 2021 Mar 25;11:6867. doi: 10.1038/s41598-021-85543-5 (PMC7994314; doi:10.1038/s41598-021-85543-5)
Supplement: Supplementary file 6 — Supplementary Information 2. [file 41598_2021_85543_MOESM6_ESM.pdf]

A 3D Analysis of Growth Trajectory and Integration During Early Human Prenatal Facial Growth

Motoki Katsube*[1], Shigehito Yamada[2, 3], Natsuko Takahashi[1,2], Yutaka Yamaguchi[3], Tetsuya Takakuwa[3], Akira Yamamoto[4], Hirohiko Imai[5], Atsushi Saito[6]; Siddharth R Vora[7], Naoki Morimoto[1]

[1]Department of Plastic and Reconstructive Surgery, Kyoto University Graduate School of Medicine, 54 Kawahara-cho, Shogoin, Sakyo-ku, Kyoto 606-8507, Japan

[2]Congenital Anomaly Research Center, Kyoto University Graduate School of Medicine, Yoshida-Konoe-cho, Sakyo-ku, Kyoto 606-8501, Japan

[3]Human Health Sciences, Kyoto University Graduate School of Medicine, 53 Shogoin-Kawahara-cho, Sakyo-ku, Kyoto 606-8507, Japan

[4]Department of Diagnostic Imaging and Nuclear Medicine, Kyoto University Graduate School of Medicine, 54 Kawahara-cho, Shogoin, Sakyo-ku, Kyoto 606-8507, Japan

[5]Department of Systems Science, Kyoto University Graduate School of Informatics, Yoshida-Honmachi, Sakyo-ku, Kyoto 606-8501, Japan

[6]Institute of Engineering, Tokyo University of Agriculture and Technology, 2-24-16 Naka-cho, Koganei-shi, Tokyo 184-8588, Japan

[7]Oral Health Sciences, University of British Columbia, JBM 372-2199 Wesbrook Mall, Vancouver, BC, V6T 1Z3, Canada

Corresponding author

Motoki Katsube, MD, PhD

Department of Plastic and Reconstructive Surgery, Kyoto University Graduate School of Medicine

54 Kawahara-cho, Sakyo-ku, Kyoto 606-8507, Japan

Phone: +81-75-751-3613

Fax: +81-75-751-4621

Email: katsube@kuph.kyoto-u.ac.jp

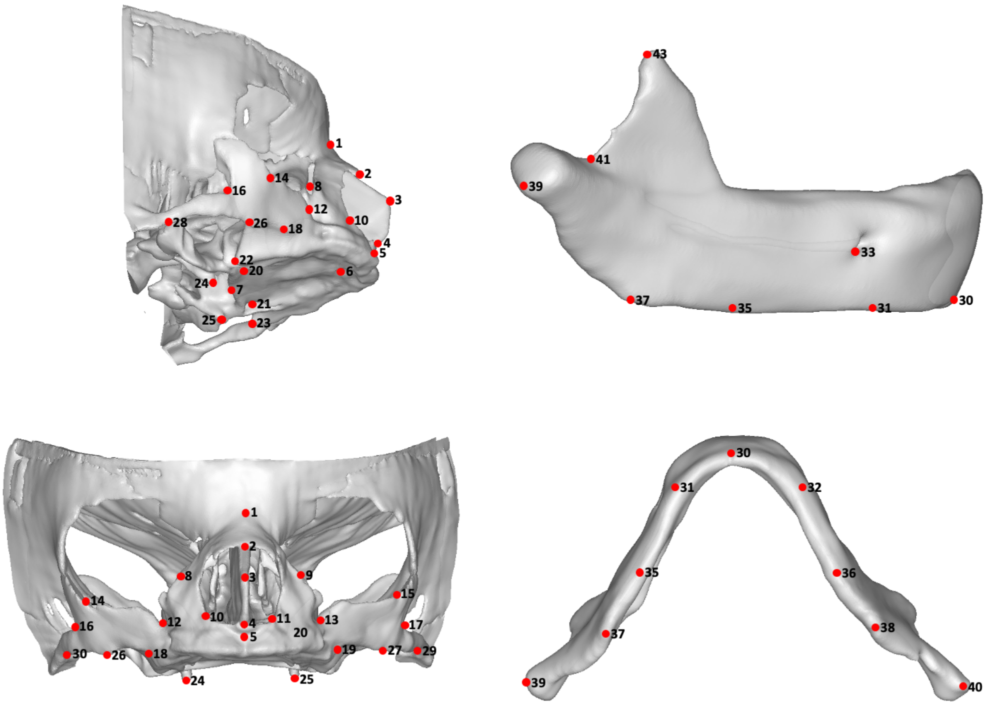

Supplementary Figure 1 Landmark location.

A total of 44 landmarks were annotated on each specimen, including 29 landmarks on the midface and 15 landmarks on the mandible (see Supplementary Table 2 for landmark definitions).

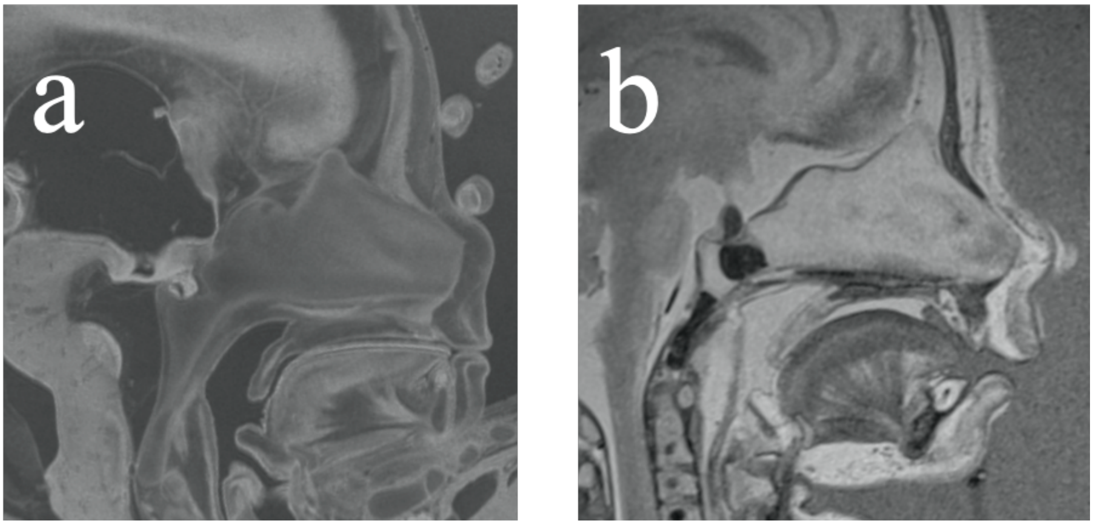

Supplementary Figure 2 Mid-sagittal images of MRI of close-mouth and open-mouth specimens. Within our specimens, there was a variation in whether the tongue contacted the palate and the lip were closed. Figure shows and example of a specimen with the tongue in contact with the palate and the lips contacting each other (**a,** Supplementary Table 1, No 23) and another specimen with the tongue not touching the palate and the lips apart (**b,** Supplementary Table 1, No 44).

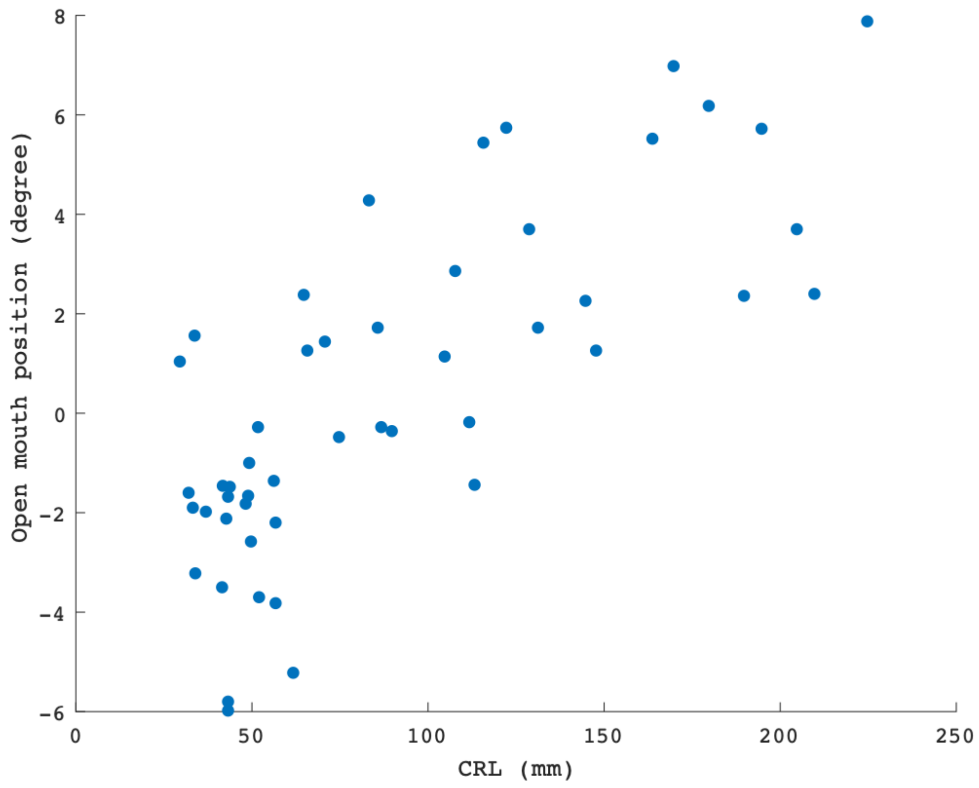

Supplementary Figure 3 Open mouth degree of each specimen.

Plot shows the open-mouth degree of each sample (y-axis) and its CRL on the x-axis. This degree was calculated when we performed the steps for correction of the mouth open position as described in Materials and Methods and outlined in Figure 2. Positive and negative values represent the rotation applied to each set of mandibular landmarks during the "rotation" step in Figure 2. As can be seen, the mouth-open degree increases with an increase in CRL. However, some of this may include an artificial mouth-opening either at the time of collection or due to fetal movement.

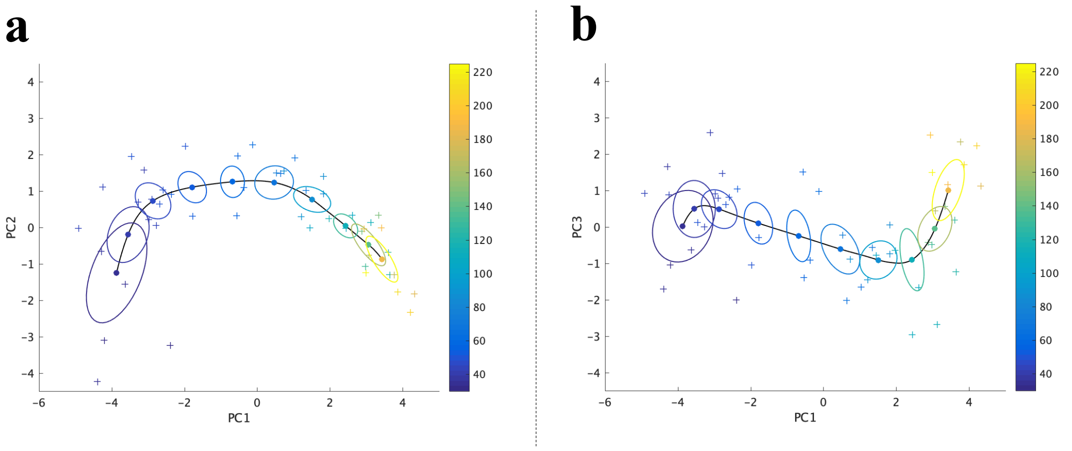

Supplement Figure 4 95% confidence region of the estimated growth trajectory.

The non-linear estimated growth trajectory was visualized in PC space 1 v/s 2 (a) and 1 v/s 3 (b). Each specimen is represented by a "+" and its color indicates its size (color bar = range of CRL). A bootstrap procedure was performed with 1000 resamples, and each ellipses represent the 95% confidence region of 10 equally spaced points on the growth trajectory.

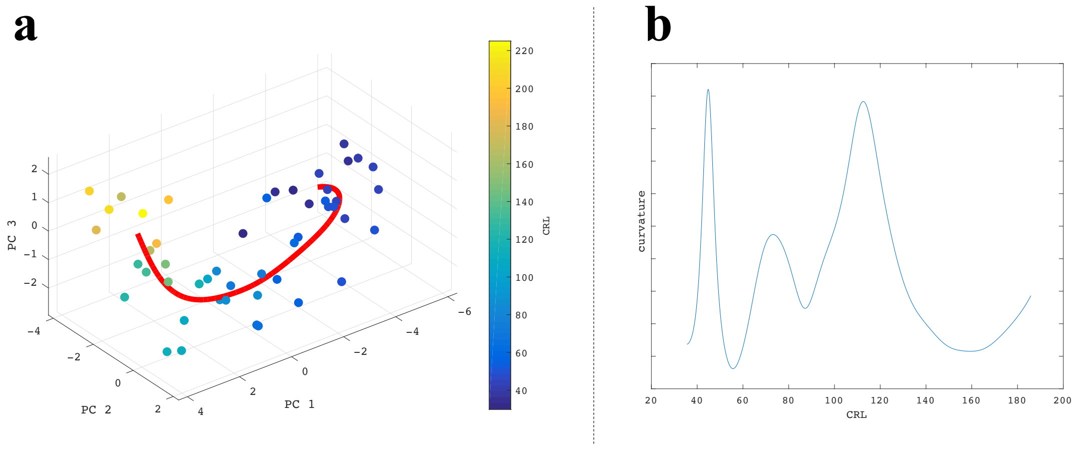

Supplementary Figure 5. The non-linear growth trajectory estimation.

The non-linear estimated growth trajectory was visualized in PC spaces 1-3 (a). Each dot represents a single specimen and its color indicates its CRL (color bar). The trajectory had 3 bending points (b), indicating that the facial morphology changes could be viewed as 4 unique segments, during this age period. Based on the CRL of specimens (44.8, 73.2 and 112.6 mm), these changes were estimated to occur at 11.3, 13.4 and 15.7 weeks of gestation.

Supplementary Table 1

Crown rump length (CRL), sex, and mouth-opening condition of specimens

No.	CRL	Sex	Tongue contact with palate	Lip closure
**1**	29.8	I	-	
**2**	32.3	I	-	
**3**	33.5	I	-	-
**4**	34	I	-	-
**5**	34.2	F		
**6**	37.2	I	-	-
**7**	41.8	M	-	
**8**	42	I		
**9**	43	I	-	
**10**	43.5	M	-	
**11**	43.5	F	-	
**12**	43.5	I	-	
**13**	44	M	-	-
**14**	48.5	M	-	
**15**	49.2	M	-	
**16**	49.5	F	-	
**17**	50	F		
**18**	52	F	-	-
**19**	52.3	M		-
**20**	56.5	F	-	
**21**	57	F	-	
**22**	57	M		
**23**	62	M		
**24**	65	F		
**25**	66	F		
**26**	71	M		
**27**	75	F		
**28**	83.5	M	-	-
**29**	86	F	-	

**30**	87	F	-	-
**31**	90	F		
**32**	105	F		
**33**	108	M		
**34**	112	M		-
**35**	113.5	F		
**36**	116	F		-
**37**	122.5	M	-	
**38**	129	F		
**39**	131.5	M		
**40**	145	M		-
**41**	148	M		
**42**	164	M		
**43**	170	F		
**44**	180	F	-	-
**45**	190	F		
**46**	195	M		
**47**	205	M		
**48**	210	M	-	-
**49**	225	M	-	

M: Male, F: Female, I: Indistinguishable

Supplementary Table 2 List of 44 Facial Landmarks with Descriptions

No	Landmark	Description
**Mid-face landmarks**		
1	Nasion	Most concave point on the midline between nasal bone and frontal bone
2	Rhinion	Midline point at the inferior end of the internasal suture
3	Nasal septum (anterior)	Most anterior point of the nasal septum
4	Anterior nasal spine	Most superior anterior midline point of the anterior nasal spine
5	Subspinale	
6	Incisive fossa	Most inferior, anterior point of the margin of the incisive fossa
7	Nasal septum (inferior posterior)	Most inferior posterior point of the nasal septum
8, 9	Nasolacrimal duct	Most superior medial point of the margin of the nasolacrimal duct
10, 11	Alare	Most inferior lateral point on the margin of the nasal aperture
12, 13	Infraorbital foramen	Most inferior anterior point of the margin of the infraorbital foramen
14, 15#	Orbital rim (lateral)	Most anterior lateral point of the inferior orbital rim
16, 17#	Jugale	Point in the depth of notch between the temporal and frontal processes of the zygomatic bone
18, 19#	Root of zygomatic process	Most inferior, anterior, medial point of the zygomatic process
20, 21	Greater palatine foramen	Most inferior, posterior, medial margin of the greater palatine foramen
22, 23	Posterior alveolar	Most posterior point of the alveolar process on the inferior surface of the maxilla
24, 25	Hamlus of the medial pterygoid plate	Most inferior point of the Hamulus of the medial pterygoid plate
26, 27#	Anterior attachment of the masseter muscle to the zygoma	Most anterior point of the attachment of the masseter muscle to the zygoma
28, 29#	Posterior attachment of the masseter muscle to the zygoma	Most posterior point of the attachment of the masseter muscle to the zygomatic arch
**Mandible landmarks**		
30	Gnathion	Most inferior anterior point of the mandibular symphysis
31, 32	Mental tubercle	Most inferior anterior point of the mental tubercle
33, 34	Mandibular foramen	Most inferior posterior point of the mental foramen

35, 36*	Anterior attachment of the masseter muscle to the mandible	Most anterior point of the attachment of the masseter muscle to the mandible
37, 38*	Gonion	Most inferior posterior point of the masseter muscle attachment
39, 40*	Condyle	Most posterior lateral point of the condylar process
41, 42*	Mandibular notch	Point in the depth of the mandibular notch
43, 44*	Coronion	Most superior posterior point of the coronoid process

#assigned as the zygoma

*assigned as the ramus of the mandible

Supplementary Table 3

MANOVA of facial skeleton against sex

	Df	Pillai	Approx F	Num Df	Den Df	Pr (>F)
Sex	1	0.44714	0.8939	19	21	0.5947
residuals	39					

Df: Degrees of freedom, Num Df: Numerator degrees of freedom, Den Df: Denominator degrees of freedom

Supplementary Text 1

Let $\boldsymbol{x}_i \in \mathbb{R}^d$ and $t_i \in [t_{\min}, t_{\max}]$ denote a feature vector and a timepoint for the i-th subject ($i \in \{1,2,\dots,n\}$). The average growth curve was estimated with the modified Nadaraya–Watson kernel estimator:

$$\widehat{\boldsymbol{x}}(t) = \frac{\sum_{i=1}^{n} K_\sigma\left(g_h(t) - g_h(t_i)\right)\boldsymbol{x}_i}{\sum_{i=1}^{n} K_\sigma\left(g_h(t) - g_h(t_i)\right)}.$$

Here K_σ is a Gaussian kernel with a bandwidth σ, and g_h is the kernel-smoothed cumulative distribution function,

$$g_h(t) = \frac{1}{n} \cdot \sum_{i=1}^{n} \int_{-\infty}^{t} K_h(t' - t_i)\, dt' \cdot (t_{\max} - t_{\min}) + t_{\min},$$

which was introduced to achieve stability against an inhomogeneous distribution of the timepoints.
